# Low-dose plasmid DNA treatment increases plasma vasopressin and regulates blood pressure in experimental endotoxemia

**DOI:** 10.1186/1471-2172-13-59

**Published:** 2012-11-08

**Authors:** Thiago Malardo, Marcelo E Batalhão, Ademilson Panunto-Castelo, Luciana P Almeida, Everton Padilha, Isabela C Fontoura, Célio L Silva, Evelin C Carnio, Arlete AM Coelho-Castelo

**Affiliations:** 1 Department of Biochemistry and Immunology, School of Medicine of Ribeirao Preto, University of São Paulo, Ribeirao Preto, SP 14049-900, Brazil; 2 Department of General and Specialized Nursing, College of Nursing of Ribeirao Preto, University of São Paulo, Ribeirao Preto, SP 14040-902, Brazil; 3 Department of Biology, School of Philosophy, Sciences and Literature of Ribeirao Preto, University of Sao Paulo, Ribeirao Preto, SP 14040-901, Brazil

**Keywords:** Endotoxemic shock, Interleukin-6, Naked pcDNA3

## Abstract

**Background:**

Although plasmid DNA encoding an antigen from pathogens or tumor cells has been widely studied as vaccine, the use of plasmid vector (without insert) as therapeutic agent requires further investigation.

**Results:**

Here, we showed that plasmid DNA (pcDNA3) at low doses inhibits the production of IL-6 and TNF-α by lipopolysaccharide (LPS)-stimulated macrophage cell line J774. These findings led us to evaluate whether plasmid DNA could act as an anti-inflammatory agent in a Wistar rat endotoxemia model. Rats injected simultaneously with 1.5 mg/kg of LPS and 10 or 20 μg of plasmid DNA had a remarkable attenuation of mean arterial blood pressure (MAP) drop at 2 hours after treatment when compared with rats injected with LPS only. The beneficial effect of the plasmid DNA on MAP was associated with decreased expression of IL-6 in liver and increased concentration of plasma vasopressin (AVP), a known vasoconstrictor that has been investigated in hemorrhagic shock management. No difference was observed in relation to nitric oxide (NO) production.

**Conclusion:**

Our results demonstrate for the first time that plasmid DNA vector at low doses presents anti-inflammatory property and constitutes a novel approach with therapeutic potential in inflammatory diseases.

## Background

Plasmid DNA has been successfully used as preventive or therapeutic DNA vaccines in experimental models of viral, bacterial or parasitic diseases [1]. Such vaccines are composed of an antigen-encoding gene, in which its expression is regulated by a strong mammalian promoter expressed on a plasmid backbone of bacterial DNA [2-4]. Although there has been a reasonable excitement about DNA vaccines because of protection induced by strong T helper 1 responses, it has become apparent that these attained responses in non-human primates and humans are weaker than those in mice, probably because of questions related to dosage and CpG stimulations [5,6]. This aspect is an important issue to improve DNA vaccines. We have previously studied DNA vaccine biodistribution [7] and also showed that naked plasmid DNA at low doses inhibits antigen presentation to T cells [8]. Besides, preliminary data from our group has suggested that naked plasmid DNA at low doses has anti-inflammatory properties.

In the last few years, Gram-negative bacteria have re-emerged as one of the most important pathogens that induce blood stream infections [9]. These blood-borne bacteria can produce serious systemic reactions, known as severe sepsis and shock septic, which are associated with marked hemodynamic alterations, such as hypotension, abnormal perfusion of organs and tissues, decreased systemic vascular resistance and increased heart rate (HR) [10]. Patients with severe sepsis have frequently presented dysfunction/failure of at least one organ and, in about 30% of the cases, multiple organ dysfunction syndrome (MODS) [11].

In Gram-negative sepsis, hemodynamic instability in response to infection is due to excessive production of inflammatory mediators by endotoxin-activated inflammatory cells [12]. Endotoxins, such as outer membrane wall component lipopolysaccharide (LPS) from Gram-negative bacteria [13], are responsible for direct activation of cells and indirect inflammatory cascade, leading to the production of tumor necrosis factor-α (TNF-α, interleukin (IL-) 1, IL-6 and IL-8 by macrophages and monocytes [14-17]. The production of these pro-inflammatory cytokines is dependent on LPS-binding protein, receptors CD14 and Toll-like receptor (TLR) 4 [18] and following the intracellular activation cascade, which triggers the nuclear translocation of nuclear factor-κB (NF-κB) and following activation of cytokines gene promoters [19].

IL-6, TNF-α and other inflammatory cytokines are of fundamental importance in sepsis development by mediating some biological responses, such as elevated production of nitric oxide (NO) by macrophages [20]. NO is a highly diffusible gas that is produced through a nitric oxide synthase (NOS)-catalyzed oxidation of l-arginine to l-citrulline. Although NOS presents non-inducible isoforms, it is the inducible NOS (iNOS) isoform, present in activated leukocytes, that contributes to vascular hyporesponsiveness and hemodynamic alterations associated with sepsis [21]. Moreover, iNOS-deficient mice are resistant to LPS-induced death, indicating a critical role of NO in sepsis development [22].

As the major symptoms of sepsis are related to pro-inflammatory and coagulant mediators, many studies have focused on molecules that could modulate these mediators. However, treatment of severe sepsis and septic shock with corticosteroids, the most powerful anti-inflammatory agents, has been controversial [23]. Besides, clinical experience with mediator-specific anti-inflammatory agents in sepsis has been disappointing [24]. The fact that preliminary data from our group suggested that naked plasmid DNA at low doses might have anti-inflammatory properties motivated us to evaluate plasmid DNA in experimental endotoxemia. Here, we demonstrated that low dose of plasmid DNA can decrease inflammatory cytokines and the initial hypotension noticed in endotoxemia, opening new perspectives for the treatment of inflammatory diseases.

## Methods

### Ethical approval

All experiments were performed in accordance with institutional ethical guidelines of the Animal Care Committee of the University. Approval was granted by the Committee of the University of Sao Paulo at Ribeirão Preto School of Medicine (CETEA-FMRP-USP).

### Rats and reagents

Experiments were performed on adult male Wistar rats weighing 250–300 g at the time of surgery. The animals were maintained under standard conditions with controlled temperature (25.0 ± 2°C) and exposed to a daily 12:12 h light dark cycle, in the animal house of the University of São Paulo at Ribeirão Preto College of Nursing, Ribeirão Preto, SP, Brazil.

RPMI 1640 medium, Hepes, fetal calf serum (FCS), TRIzol and plasmid pcDNA3 were obtained from Invitrogen (Carlsbad, CA, USA), and L-glutamine, 2-mercaptoethanol, penicillin, streptomycin, lipopolysaccharide (LPS) from *Escherichia coli* serotype 0111:B4, dexamethasone and 2,2,2-tribromoethanol, sodium nitrate were obtained from Sigma-Aldrich Co. (St. Louis, MO, USA). Cell culture plates were purchased from Corning Inc. (Corning, NY, USA). Recombinant mouse IL-6, capture and biotinylated monoclonal anti-IL-6 (clones MP5-20F3 and MP5-32C11) and an OptEIA mouse TNF-α (Mono/Mono) set were purchased from BD Biosciences (San Jose, CA, USA). The forward and reverse primers of IL-6 (f: TCCTACCCCAACTTCCAATGCTC and r: TTGGATGGTCTTGGTCCTTAGCC), TNF-α (f: AAATGGGCTCCCTCTCATCAGTTC and r: TCTGCTTGGTGGTTTGCTACGAC) and β-actin (f: AAGTCCCTCACCCTCCCAAAAG and r: AAGCAATGCTGTCACCTTCCC) were purchased from Invitrogen (São Paulo, Brazil).

### Plasmid DNA preparation

pcDNA3 was propagated in *Escherichia coli* strain DH5α and large-scale preparations of plasmid DNA were carried out using the EndoFree Plasmid Giga Kit (Qiagen, Ltd., Crawley, UK) according to the manufacturer’s instructions. Spectrophotometric analysis revealed 260/280 nm ratios ≥ 1.80. The purity of DNA preparations was confirmed on a 1% agarose gel. Samples of plasmid in the doses described below were administered in normal rats and we observed no changes in body temperature, suggesting absence or very low concentration of endotoxin.

### Cell line culture

The macrophage cell line J774 (5 × 10^5^ cells/mL) was suspended in complete RPMI medium (RPMI 1640 containing 2 mM L-glutamine, 50 μM 2-mercaptoethanol, 100 units/ml penicillin, 100 μg/mL streptomycin and 5% heat-inactivated FCS), seeded in 24-well cell culture plates, 1 mL per well, and stimulated with plasmid pcDNA3 at the concentration ranging from 1 to 100 μg/mL. In the inhibition experiments, J774 cells were simultaneously stimulated with 500 ng of LPS and pcDNA3 at the concentration of 3, 5 or 10 μg/mL. LPS (500 ng) was also used as the positive control. After incubation at 37°C in a humidified 5% CO_2_ atmosphere for 48 h, the supernatants were harvested and the concentrations of IL-6 and TNF-α were determined by ELISA, according to recommendations obtained from BD Biosciences.

### Experimental design

Rats were submitted to general anesthesia with intraperitoneal injection of 2.5% 2,2,2-tribromoethanol (10 mL/kg body weight) and implanted with a polyethylene catheter in the femoral artery for direct blood pressure recording and in the jugular vein for intravenous (i.v.) drug administration. The animals were injected i.v. by bolus injection with 1.5 mg LPS/kg body weight in a final volume of 0.5 mL of pyrogen-free sterile physiological saline (0.15 M NaCl). Experimental time “zero” was determined as the moment when LPS was injected. Control rats were injected i.v. with 0.5 mL sterile saline.

In a separate set of experiments, rats received an i.v. injection of 5, 10 or 20 μg plasmid DNA in a final volume of 0.5 mL physiological saline. Control animals were injected with the same volume of saline. Two minutes later, animals were injected with LPS as described above.

Mean arterial blood pressure (MAP) and HR of anaesthetized freely moving rats were recorded using a polygraph (Grass P122), connected to a pressure transducer (Grass P23XL-1) and using the software PolyView (Astro-Med, West Warwick, RI, USA), over a period of 4–6 h after LPS or saline i.v. injection. The rats were decapitated 2, 4 and 6 h after LPS or saline administration. Blood samples were collected into chilled heparinized plastic tubes, centrifuged for 20 min at 2000 × g at 4°C. Plasma samples were stored at −70°C before dosage. Liver samples were excised and frozen in liquid nitrogen and stored at −70°C until processing.

### Plasma NO and arginine vasopressin (AVP) dosage

On the day of the assay, plasma samples were thawed and deproteinized with 95% ethanol (at 4°C) for 30 min, subsequently centrifuged, and the supernatant was used for measurement of nitrate according to the NO/ozone chemiluminescence technique [25], using a Sievers NO Analyzer 280 (GE Analytical Instrument, Boulder, CO, USA). Sodium nitrate was used as standard reference. AVP extracted from 0.7-1.5 mL of plasma using acetone and petroleum ether was dried and stored at −20°C until radioimmunoassay measurements were performed as previously described [26].

### Real-time reverse transcription polymerase chain reaction (RT-PCR)

Total RNA from liver homogenized manually under liquid nitrogen with a mortar and pestle was extracted using TRIzol. Quantification of RNA was carried out on a NanoDrop ND1000 spectrophotometer (Fisher Thermo, Wilmington, USA) and its concentration adjusted to 0.25 μg/μL using RNase free water. Reverse transcription (RT) was performed using the Rotor-Gene 6000 Real-Time PCR machine (Corbett Life Science, Mortlake, Australia). Gene expression was determined in mRNA as previously reported [27], using primers described above.

### Data analysis

Results are expressed as means ± standard deviation. Statistical determinations of the difference between means of experimental groups were performed using one-way analysis of variance (analysis of variance) followed by the Tukey multiple comparisons test. Differences that provided *p* < 0.05 were considered to be statistically significant.

## Results

### Low dose of plasmid DNA inhibits production of IL-6 and TNF-α by LPS-stimulated macrophages

**Figure 1:**
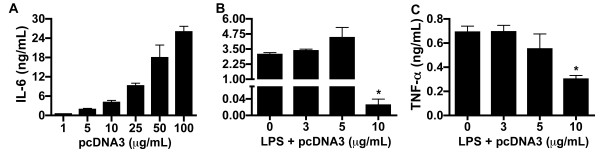
**Low-dose plasmid DNA inhibits production of IL-6 and TNF-α by LPS-stimulated macrophage cell line.** J774 cells were stimulated with pcDNA3 at different concentrations in the absence (**A**) or presence of 0.5 μg/mL of LPS (**B** and **C**). After 48 h, the concentrations of IL-6 (**A** and **B**) and TNF-α (**C**) were determined in the culture supernatants. Assays were performed in triplicate and the results represent the mean ± SD of at least three independent experiments. *, P < 0.05 versus group stimulated with LPS only.

To evaluate the production of inflammatory cytokines *in vitro* by plasmid DNA-stimulated macrophages, we incubated J774 cells with plasmid pcDNA3. Although plasmid induced the production of IL-6 in a dose-dependent manner (Figure 1A), we observed that pcDNA3 in the concentration of 10 μg/mL led to production of low concentrations of IL-6 even when LPS (0.5 μg/mL) was simultaneously used in the stimulation of the cells (Figure 1B). This suppressive effect of 10 μg/mL of plasmid on production of IL-6 by LPS-stimulated macrophage was surprising and remarkable, since there was a 99% reduction of its production when compared with cells cultured with LPS only or LPS and plasmid at the concentration of 3 or 5 μg/mL (Figure 1B). TNF-α production by LPS-stimulated J774 cells was also significantly suppressed with 10 μg/mL of pcDNA3 (Figure 1C).

### Low dose of plasmid DNA attenuates the LPS effect on the experimental endotoxemia

**Figure 2:**
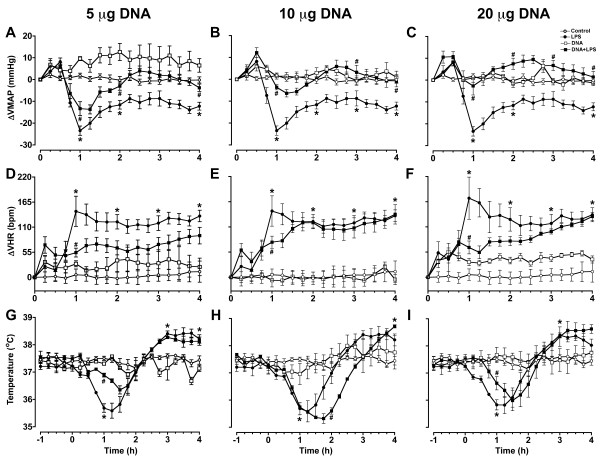
**Effect of administration of plasmid DNA on the alterations of MAP, HR and body temperature triggered by LPS in rats.** Animals were injected with LPS and plasmid DNA at doses of 5, 10 or 20 μg. Mean arterial blood pressure (ΔMAP) (**A**, **B** and **C**), variability of heart rate (ΔVHR) (**D**, **E** and **F**) and body temperature (**G**, **H** and ***I***) were monitored continuously. Results are expressed as mean ± SD of at least two independent experiments. *, P < 0.05 versus control group injected only with physiologic saline. #, P < 0.05 versus group injected with LPS only.

Since plasmids was found to decrease *in vitro* production of TNF-α and IL-6 by LPS-stimulated macrophage cell line and these cytokines play a key role in the inflammatory process, we evaluate if low doses of plasmid could have a beneficial effect *in vivo* in an endotoxemia model. To validate our experimental model, first we determined the MAP, HR and plasma concentration of NO and AVP in LPS-injected rats. As expected, the animals injected with LPS had a significant decrease in MAP and increase in HR, plasma nitrate and AVP concentrations as compared to control rats (Figure 2). When low doses of plasmid DNA were injected almost simultaneously (2 minutes before) with LPS, we observed that the administration of 10 or 20 μg of plasmid significantly attenuated the MAP drop (Figure 2B and C). The plasmid treatment did not have considerable effect on the alterations of HR and body temperature induced by LPS (Figure 2D-I).

**Figure 3:**
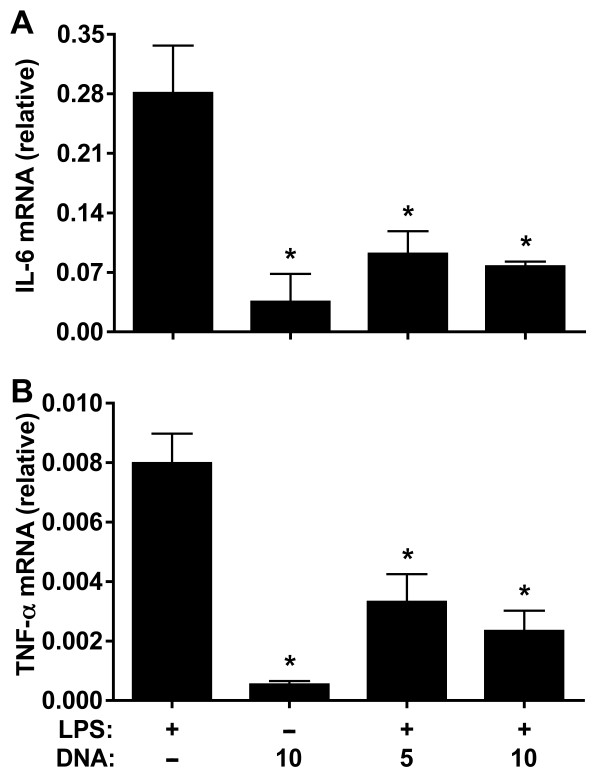
**Administration of DNA in rats injected with LPS decreases liver expression of IL-6 and TNF-α.** Animals were injected with LPS and plasmid DNA at doses of 5 or 10 μg. At 2 (**A**) or 4 (**B**) hours after injection of LPS, relative amounts of mRNA for IL-6 (**A**) and TNF-α (**B**) from liver were determined. Results are expressed as mean ± SD of at least two independent experiments. *, P < 0.05 versus control group injected with LPS only.

**Figure 4:**
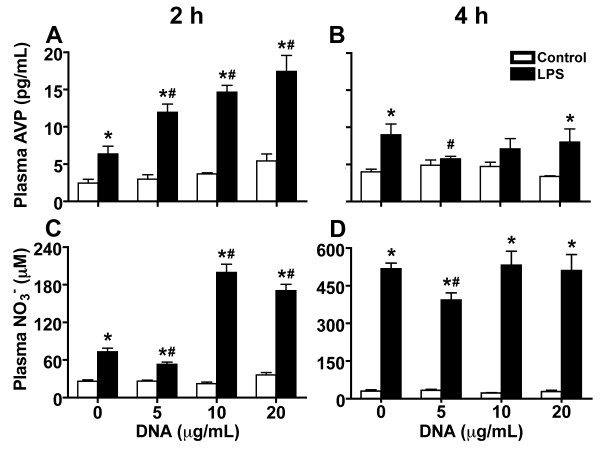
**Effect of plasmid DNA on plasma concentration of NO**_**3**_^**-**^**and AVP in LPS-injected rats.** Animals were injected with LPS and plasmid DNA at doses of 5, 10 or 20 μg. At 2 (**A** and **C**) and 4 (**B** and **D**) hours after injection of LPS, blood was collected and the concentration of plasma AVP (**A** and **B**) and NO_3_^-^ (C and D) was determined. Results are expressed as mean ± SD of at least two independent experiments. *, P < 0.05 versus control group injected only with physiologic saline. #, P < 0.05 versus group injected with LPS only.

To explain the mechanism involved in the plasmid-induced attenuation of MAP drop in LPS-stimulated rats, we determined the gene expression of liver IL-6 and TNF-α and plasma concentration of AVP and NO, key mediators in endotoxemia. Similar to *in vitro* experiments, low doses of plasmid DNA (5 and 10 μg) induced a significant decrease of TNF-α and IL-6 message in the livers of LPS-injected rats. IL-6 was diminished at 2 hours after LPS injection when compared with the rats administered with LPS only (Figure 3), whereas no significant difference was detected at 4 hours after LPS injection (data not shown). In contrast, low doses of plasmid DNA induced a significant reduction in the TNF-α at 4 hours of experiment, but not at 2 hours, when compared with the group treated with LPS only (Figure 3). As the fall in MAP occurs in the first two hours after injection of LPS, we suggested that the decrease of IL-6 may be important in reducing the pressure drop observed in animals treated with plasmid DNA. Like IL-6, two hours after LPS administration, we observed that all tested dose of plasmid DNA significantly enhanced the LPS effect on AVP production, whereas only the doses of 10 and 20 μg of plasmid led to a significant increase in NO (Figure 4). Interestingly, the concentrations of AVP were low after 4 h of LPS injection, whereas high concentrations of NO were maintained (Figure 4).

## Discussion

In this research, we demonstrated for the first time that a low dose of plasmid DNA vector induces the production of low concentration of inflammatory cytokine IL-6 and TNF-α by *in vitro* LPS-stimulated macrophages. The novelty of this contribution lies in the fact that we evaluated this inflammatory suppression by a non-coding plasmid DNA in an endotoxemia model in rats and obtained a remarkable attenuation in the drop of MAP.

Since the sharp fall in MAP in case of endotoxemia can be a life-threatening situation, the maintenance of MAP is one of the major concerns in its treatment [28]. Our data demonstrated that plasmid DNA, mainly at doses of 10 and 20 μg, when administered concomitantly with LPS in rats, significantly prevented the drop in MAP within the first hour of the experiment. This stabilization of MAP was maintained until the fourth hour after LPS injection. Although we cannot exclude that there may have been *in vivo* direct interaction between plasmid and LPS, this seems unlikely because this interaction should induce an inhibition of all parameters analyzed in a dose response manner. Our *in vivo* experiments demonstrated that 5 μg of plasmid was able to produce a decrease of the effect of LPS on the temperature of rats, but not on the ΔVHR and ΔVMAP, when compared with the doses of 10 and 20 μg. In contrast, 10 and 20 μg of plasmid diminished the effect of LPS on the ΔVHR and ΔVMAP, but not on temperature.

AVP and NO have been described as important mediators in septic shock [29-32]. AVP is a hormone that raises blood pressure by regulating vascular water balance and inducing vasoconstriction [33]. The plasma concentration of AVP is increased in the first and second hour after administration of LPS in rats [34], as seen in our control 2 hours after LPS injection. More importantly, all tested doses of plasmid DNA were able to further increase the concentration of plasma AVP in LPS-injected rats at the second hour of experiment, in a dose dependent manner. Though plasmid DNA was responsible for the increase of AVP in the first 2 hours, its effect was indirect, since rats injected with only plasmid DNA did not have increased AVP concentrations. This increase in plasma AVP concentrations suggests it could be responsible for recovering MAP.

Concerning to NO, although some studies have demonstrated that specific iNOS inhibitors can reduce the MAP drop in animal models [34-36], this has not been observed in septic patients [37,38]. Even so, we analyzed NO because it is considered one of the major factors responsible for refractory hypotension in sepsis, has its concentration increased after LPS injection [39], and appears to play a key inhibitory role in AVP released during endotoxemia, leading to hypotension [34]. Apparently, NO over-production from endotoxin/cytokines-induced iNOS mediates a vasodilatation difficult to revert with vasoconstrictors [40]. In our experiments, NO level was decreased until the fourth hour of the experiment only in the group administered with LPS and treated with 5 μg of plasmid DNA. Interestingly, 5 μg of plasmid DNA was not able to maintain the MAP in these rats. In contrast, 10 and 20 μg of plasmid DNA, which best stabilized the MAP in the second hour after LPS administration, were the doses that significantly increased the concentrations of NO when compared to rats injected with LPS only. Therefore, even with high production of NO, the LPS-injected rats treated with plasmid DNA did not show a drop in MAP.

Given that physiological changes in sepsis are in part caused by high concentrations of inflammatory mediators, it is reasonable to suppose that, among the treatments that have been investigated in sepsis, emphasis has been given to the inhibition of these mediators [41]. In our experiment *in vitro*, plasmid DNA significantly decreased the production of IL-6 and TNF-α by LPS-stimulated macrophage cell line J774. The effect of plasmid DNA on LPS-stimulated J774 cells was more pronounced on the IL-6 than TNF-α, i.e., 99% reduction for IL-6 and 56% for TNF-α when compared with macrophages stimulated with LPS only. Similar results were obtained when our *in vitro* experiments were done with another plasmid (pVAX), suggesting that the anti-inflammatory property does not appear to be restricted to the pcDNA3 (data not shown). Because studies have indicated that Kupffer cells promote a significant role in the production of proinflammatory cytokines, such as TNF-α, IL-1 and IL-6, after stimulation with LPS [42-44], we evaluated IL-6 and TNF-α messages from liver. Interestingly, the results of real time RT-PCR showed that the *in vivo* gene expression of IL-6, but not TNF-α, was decreased significantly at 2 hours after plasmid DNA and LPS injection when compared with injection of LPS only, suggesting that IL-6 contributes to the stabilization of MAP. Such stabilization was not attributed to the TNF-α as it was decreased only at 4 hours after plasmid DNA and LPS injection. The role of IL-6 in murine sepsis has been somewhat controversial, since data from the literature are conflicting in relation to survival of IL-6 knockout mice subject to cecal ligation and puncture [45-47]. Nevertheless, IL-6 has pointed out as an important cytokine in sepsis with pro- and anti-inflammatory effects [48,49], regardless of whether it is a disease biomarker or contributes to severity of sepsis [50]. More importantly, IL-6 appears to be one of the best predictors to determine MODS, sepsis severity and mortality in both animal models [51,52] and human [49,53-56]. The former data from the literature supported our hypothesis that low expression of IL-6 induced by the plasmid DNA treatment was crucial to dampen the LPS-induced hypotension in rats.

AVP infusion has been suggested as an alternative therapy for septic shock patients that are refractory to usual vasopressor therapy [29]. This therapy can be justified because plasma AVP concentrations in septic patients are maintained close to physiological level, although a lower blood pressure is noticeable [57]. Because plasma concentrations of AVP might be inappropriately low in septic shock [57] and, as seen in this study, plasmid DNA can increase concentration of AVP and improve MAP, we suggested that plasmid DNA constitutes a novel approach with therapeutic potential in sepsis that may have clinical applications.

## Conclusion

We demonstrated that rats injected concomitantly with LPS and small doses of plasmid DNA had a remarkable attenuation in the drop of blood pressure at 2 hours after treatment when compared with rats injected with LPS only. The beneficial effect of plasmid DNA on blood pressure was associated with decreased expression of IL-6 in liver and increased plasma vasopressin concentrations. Our results demonstrate for the first time that plasmid DNA vector can be anti-inflammatory and constitute a novel approach with therapeutic potential in inflammatory diseases.

## Competing interest

The authors declare that they have no competing interests.

## Authors’ contributions

TM, MEB, EP, ICF and LPA participated in the design of the study and experiments. AAMC-C, AP-C, CLS and ECC conceived the study and participated in its design and coordination. AP-C and AAMC-C wrote the paper. All authors read and approved the final manuscript.
